# Drug hypersensitivity syndrome induced by Beta--lectam and "Cold3" for the same patient

**DOI:** 10.1186/2045-7022-4-S3-P78

**Published:** 2014-07-18

**Authors:** Ingrida Maciulaityte, Simona Kasinskaite, Kristina Bliudziute, Violeta Kvedariene

**Affiliations:** 1 Vilnius University, Lithuania; 2 Vilnius University, Center of Pulmonology and Allergology, Lithuania; 3 Vilnius University, Center of Internal Medicine, Lithuania

## 

Drug hypersensitivity syndrome is difficult to diagnose, as many of its clinical features could imitate systemic disorders.

The aim of our study is to present the case of patient with drug hypersensitivity syndrome induced by two different kind of drugs in one year period.

A 65-year female took amoxicillin due to viral bronchitis. The second day 6-8 hours after applying the drug she developed generalized maculopapular egzanthema with itchiness. She was hospitalized in Vilnius University Hospital Santariskiu Klinikos because of progressive symptoms. The elevation of liver enzymes and discrasia were found. Specific IgE to amoxicillin, ampicillin, penicillin G, penicillin V were negative. Symptoms completely disapeared after one month of treatment. After three months patch test with amoxicillin was carried out. 72 hours later the test was positive. Six months later patient was treated with "COLD3" (paracetamol, pseudoephedrine, dekstrametorfan), a cedar oil and homeopathic medications because of respiratory viral infection. The same day similar clinical picture appeared. Three month later we performed patch test with"COLD3", cedar oil and homeopathic drugs. Patch test with "COLD3", after 72 hours was positive and others - negative. Herpes simplex, EBV, CMV, and HBV, HCV were investigated and were negative.

## Conclusion

Viral infection and hypersensitivity to drugs may induce drug hypersensitivity syndrome. It is essential to perform biochemical tests for patients presenting with a high fever and extensive skin rash after the intake of drugs.

